# Mathematical modeling of the optimum pulse structure for safe and effective photo epilation using broadband pulsed light

**DOI:** 10.1120/jacmp.v13i5.3702

**Published:** 2012-09-06

**Authors:** Caerwyn Ash, Kelvin Donne, Gwenaelle Daniel, Godfrey Town, Marc Clement, Ronan Valentine

**Affiliations:** ^1^ School of Medicine Swansea University Swansea UK; ^2^ Department of Medical Physics University of Wales Cardiff UK; ^3^ Department of Medical Physics University of Dundee Dundee UK

**Keywords:** IPL, Monte Carlo modeling, pulse structure, hair removal

## Abstract

The objective of this work is the investigation of intense pulsed light (IPL) photoepilation using Monte Carlo simulation to model the effect of the output dosimetry with millisecond exposure used by typical commercial IPL systems. The temporal pulse shape is an important parameter, which may affect the biological tissue response in terms of efficacy and adverse reactions. This study investigates the effect that IPL pulse structures, namely free discharge, square pulse, close, and spaced pulse stacking, has on hair removal. The relationship between radiant exposure distribution during the IPL pulse and chromophore heating is explored and modeled for hair follicles and the epidermis using a custom Monte Carlo computer simulation. Consistent square pulse and close pulse stacking delivery of radiant exposure across the IPL pulse is shown to generate the most efficient specific heating of the target chromophore, whilst sparing the epidermis, compared to free discharge and pulse stacking pulse delivery. Free discharge systems produced the highest epidermal temperature in the model. This study presents modeled thermal data of a hair follicle *in situ*, indicating that square pulse IPL technology may be the most efficient and the safest method for photoepilation. The investigation also suggests that the square pulse system design is the most efficient, as energy is not wasted during pulse exposure or lost through interpulse delay times of stacked pulses.

PACS number: 87.10.Rt

## I. INTRODUCTION

**Figure 1:**
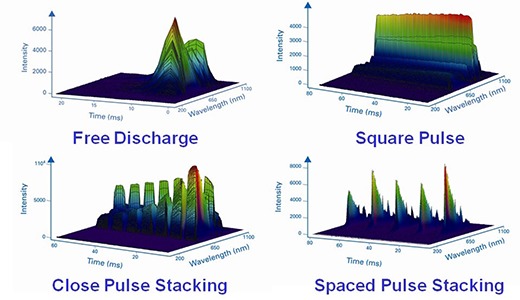
Time‐resolved spectral images of the four categories of IPL systems currently available (intensity is arbitrary).^(7)^

Various light sources have been used for photo epilation since the early 1990s such as ruby lasers,^(1)^ alexandrite lasers,^(2)^ diode lasers,^(3)^ Nd:YAG lasers,^(4)^ and intense pulsed light (IPL) systems.^(5)^ The motive for the removal of unwanted body hair can be for a number of cosmetic or medical reasons. Both men and women have a choice of many methods such as shaving, tweezing, electrolysis, threading, sugaring, depilatory lotions and creams, waxing, lasers or IPLs to remove unwanted hair.^(6)^ When clinical outcomes of IPL treatments are reported in peer‐reviewed articles, authors tend to list the manufacturer, model, fluence (radiant exposure), and overall pulse duration, but offer no details on the temporal pulse shape. Furthermore, in most cases, IPL manufacturers do not specify how the pulse is delivered (free discharge or square pulse) either in marketing materials or technical specifications given in the user manual. The temporal pulse shape is an important parameter that may affect the response of any biological tissue. Previously Ash et al.^(7)^ demonstrated that IPL systems can be categorized into four types, namely free discharge, square pulse, close pulse stacking, and spaced pulse stacking, as illustrated in Fig. 1.

Using a computer simulation, this study investigates the effect these categories of pulse structure have on hair removal for critical evaluation of system performance. Our challenge in mathematical modeling the broad wavelength distribution in IPL systems is the requirement to incorporate the numerous optical parameters listed in Table 1, such as absorption coefficients, scattering coefficients, and anisotropy factors on light propagation within tissues. The absorption coefficients are tissue‐ and wavelength‐dependant and thus cannot be averaged; therefore, we modeled the propagation in tissue of an IPL source simultaneously for each wavelength comprising the IPL spectrum. Mathematical models used to optimize selective photothermolysis in laser treatments have been widely published.^8^, ^12^ Monte Carlo simulations of light propagation in tissue are seen as the ‘gold standard’ to describe the transport of photons in biological turbid tissue^(13)^ and have worked satisfactorily for some years, where each photon is followed through a propagation medium and its journey is directed by probability distributions of its interaction of scattering angles and absorption within the various tissue layers. In particular, Bjerring et al.^(14)^ showed the importance of the pulse shape of a pulsed dye laser (PDL) using mathematical and clinical evidence. Steiner et al.^(15)^ showed the effect of altering pulse shape and wavelengths of a laser system for epilation, and demonstrated that pulse shape bore significance in relation to efficacy and safety. Shafirstein et al.^(16)^ presented a study in 2006 using mathematical modeling to aid vascular treatments using a dye laser, and recognized the temporal pulse shape as an important parameter affecting treatment effectiveness and safety outcomes/issues. Two recent studies have shown IPL technology to be a competitor to the laser in dermatology applications using modeling of blood vessels and hair follicles, respectively.^17^, ^18^

**Table 1:** Physical constants used for the various tissue layers used in this model.

	*k (W m* ^*‐1*^ *k* ^*‐1*^ *)*	ρ *(kg m* ^*‐3*^ *)*	*c (J kg* ^*‐1*^ *k* ^*‐1*^ *)*	*Refractive Index*	*Anisotropy Factor*
Epidermis	0.5	1200	3600	1.34	0.789
Dermis	0.53	1200	3800	1.37	0.789
Hair	0.24	1210	3500	1.7	0.789

Construction of a mathematical model for IPL related selective photothermolysis to correlate parameters, such as IPL spectrum and pulse structure, to the temperature distribution within tissue structures may help to improve the clinical treatment of various dermatological disorders in clinical practice.

***Selective photothermolysis***

Anderson and Parrish^(19)^ first introduced the principle of selective photothermolysis applicable to the laser treatment of cutaneous lesions in 1983. Selective photothermolysis describes how a target can be selectively destroyed by using the appropriate wavelength, pulse duration, and energy, and that the optimum level of thermal absorption in the target should be achieved with the least interference to the surrounding non‐target tissue.^(19)^ In tandem with the principle of selective photothermolysis is the concept of thermal relaxation time.^(20)^ Thermal relaxation time describes the time period for an object to lose 50% of its induced thermal energy to surrounding tissues. With the right combination of wavelength, energy, and pulse duration, it is therefore possible to target a hair follicle precisely without causing injury to the surrounding structures. One way to achieve greater injury to the hair follicle is by extending the pulse duration of the light exposure. The thermal relaxation time for hair follicles that are 200–300 μm in diameter is approximately 40 to 100 milliseconds. If pulse duration was the only factor, then the ideal pulse duration should lie between the thermal relaxation time for melanosomes found in the epidermis, which is approximately 3 to 10 milliseconds, and the thermal relaxation time for hair follicles.^(19)^ Applying the theory of selective photothermolysis using a laser for cutaneous disorders is the same when using IPL technology. With respect to lasers, IPL systems emit multiple wavelengths of light within the range of 500 nm to 1100 nm at different intensities.^(21)^ These could be modeled as 600 wavelengths of 1 nm bandwidth and varying radiant exposure simultaneously.

## II. MATERIALS AND METHODS

**Figure 2:**
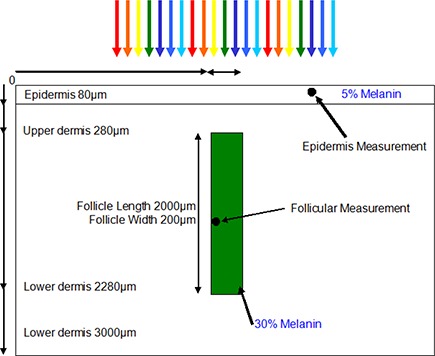
Diagram depicting the Monte Carlo Model Cartesian geometry used.

**Figure 3:**
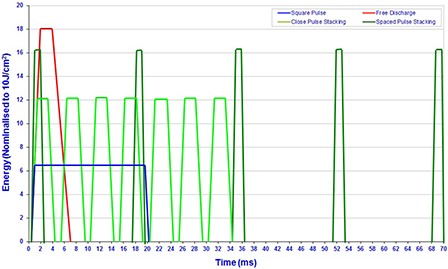
Illustration of the input pulse structures to represent the four categories of IPL systems all normalized to $10J/{\text{cm}}^{2}$ radiant exposure.

The two‐dimensional mathematical simulation described here was previously utilized for an earlier study modeling blood vessels,^(14)^ but was modified to simulate the two‐dimensional selective photothermolysis of a buried hair follicle using a typical IPL spectrum with a uniform spatial distribution. The authors of this study modeled the tissue matrix using a 5 μm cell size as a Cartesian coordinate, an 80 μm melanin rich epidermis with 5% melanin concentration, a melanin free dermis, and a buried hair follicle 200 μm below the surface of 200 μm diameter with a 30% melanin concentration extending to 2000 μm in length. The skin temperature was measured 20 μm below the skin surface; the hair follicle temperature was measured 10 μm into the side of the hair follicle at a depth of 2000 μm, as depicted in Fig. 2. The simulation is comprised of two interacting subroutines — a Monte Carlo photon distribution and a heat diffusion calculation — to compare four different pulse structures as produced by various IPL systems (shown in Fig. 3 and listed in Table 2). The model was calibrated against thermography measurements of human skin exposed to IPL exposure. These measurements used various skin tones (Caucasian, Indian, and Afro Caribbean) with ranging IPL exposure $\left(2-20\;J/{\text{cm}}^{2}\right)$ at a known distance from skin.

**Table 2:** Pulse × typically used for hair removal using four different IPL systems (values are in SI units).

	*Rise Time*	*Hold Time*	*Fall Time*	*Off Time*	*Number of Pulses*	*Energy per Pulse*
Free Discharge	0.0015	0.002	0.003	0.005	1	30.00
Square Pulse	0.0001	0.02	0.0001	0.005	1	30.00
Close Pulse Stack	0.0001	0.004	0.0001	0.001	7	4.29
Spaced Pulse Stack	0.0001	0.002	0.0001	0.015	5	6.00

**Figure 4:**
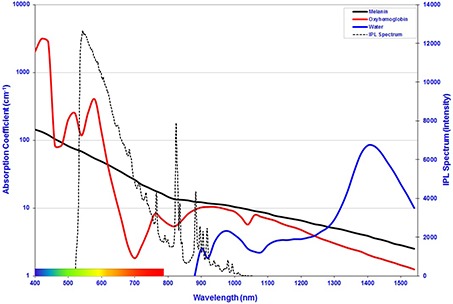
Absorption coefficients of melanin, oxyhaemoglobin, and water. The IPL emission spectrum used for this evaluation is overlaid for Reference (6).

The calculation of the energy distribution was achieved by using a Monte Carlo process. This computer simulation allows observation of comparative data that cannot be performed in skin. The Monte Carlo simulation of photons is a numerical calculation to compute the radiative transport in scattering and absorbing media. In the computations of photon pathways, the absorption coefficients $\left(\mu_{a}\right)$, the scattering coefficient $\left(\mu_{s}\right)$, and the anisotropy factor (g) of each material at the specific wavelength is considered. These respective values were taken from the literature^22^, ^23^ and the respective absorption coefficients at these wavelengths are shown in Fig. 4. The wavelength dependency of the optical parameters of the IPL spectrum complicates mathematical modeling of light tissue interaction for IPL, as compared to monochromatic lasers.^(17)^ For comparison of system design and efficiency, the total radiant exposure of the $1\times 10^{9}$ photons that constitute the spectrum shown in Fig. 4 equals $10J/{\text{cm}}^{2}$. This radiant exposure is above the published $5J/{\text{cm}}^{2}$ threshold for follicular damage.^(24)^ When filtered broadband IPL is delivered to the subject's skin, a fraction of the optical energy is reflected from the skin's surface while the rest is scattered beneath the skin's surface and is then absorbed in biological cells. Such absorption causes the hair shaft and hair bulb to heat up, resulting in damage to the follicle, shedding of the hair, and prevention of future regrowth.

A custom Monte Carlo simulation package was created by the Faculty of Design and Engineering at Swansea Metropolitan University, UK, and implemented on a personal computer. Within this Monte Carlo simulation, the absorption coefficient of melanin, $\mu_{a}$, is computed and graphically shown in Fig. 4. The scattering coefficient (cm^‐1^) is measured from experimental techniques originating from Mie scattering due to collagen fibres and from Rayleigh scattering due to small tissue structures, respectively.^(25)^ Scattering in tissue by photons is characterized by the Henyey‐Greenstein scattering phase function,^26^, ^27^, ^28^, ^29^ which is mathematically expressed in the form

(1) $$P(\theta )=\frac{1‐g^{2}}{{\left(1+g^{2}‐2\;g\;\text{Cos}(\theta )\right)}^{3/2}}$$

The longitudinal angle of scattering, (θ), is characterized by the Henyey‐Greenstein phase function and, since this phase function is forwardly biased, θ will be randomly influenced to reflect this characteristic. Applying the probability density function for the random number R to the above equation and solving for Cos(θ) leads the following expression:

(2) $$\text{Cos}(\theta )=\frac{1}{2g}\left[1+g^{2}‐{\left(\frac{1‐g^{2}}{1‐g+2\text{gR}}\right)}^{2}\right]\;\text{for}\;g\neq 0$$

**Figure 5:**
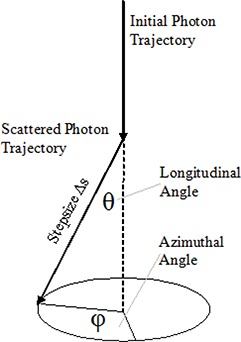
Deflection of a photon at a scattering point.

The azimuthal angle, ϕ, (Fig. 5) is uniformly distributed within the interval [0, 2π]. Its probability density function is constant and equals $\frac{1}{2\pi }$. Hence ϕ takes the form:

(3) φ=2πR

A predefined number of photons are directed onto a skin model. These photons are given a weight (W), which is equal to 1. A photon starts at a boundary position with the tissue interface with a step size, anisotropy angle of rotation, and a deflection angle. At each interaction the photon delivers a portion of its energy, preset to values determined of tissue optical properties. The loss in energy at each of the ongoing interaction sites is determined by the weight that the photon deposits. The change in weight is defined by the following equation:^30^, ^31^, ^32^

(4) $$\Delta W=W\frac{\mu_{a}}{\mu_{a}+\mu_{s}}$$

The photons are terminated when the value of ΔW is below a threshold. In this simulation the photons are terminated when one hundredth of the original weight remained after multiple interactions. Then after this photon is deposited in its final position, a new photon is released and the process is repeated. A sufficient number of photons are required to generate an absorption energy density matrix for the defined tissue configuration for absorption of the IPL power output.^30^, ^32^, ^33^ This energy density matrix is used with a factor of total energy output within this defined area.^30^, ^31^ The absorbed energy at each Cartesian cell can be used as a heat source for thermal diffusion approximation. The time dependant heat flow equation is reduced to its

2‐D form and subsequently evaluated over the discretized domain using the alternating direction implicit (ADI) method:

(5) $$\frac{\partial^{2}T}{\partial y^{2}}\_\frac{\partial^{2}T}{\partial z^{2}}+\frac{H}{k}=\frac{1\;\partial T}{\alpha \;\partial t}$$

where *H* is the volumetric distribution of energy calculated in the Monte Carlo simulation and stored in numabs array, and *y* and *z* are the radial and axial coordinates, respectively.

## III. RESULTS

The effect of delivered IPL pulse structure was studied on a buried hair follicle using the Cartesian model depicted in Fig. 2. The skin temperature was measured 20 μm below the skin surface; the hair follicle temperature was computed 10 μm into the side of the hair follicle at a depth of 2000 μm. This location is where pluripotential stem cells responsible for hair growth are thought to reside. It has been postulated that follicular damage would occur at temperatures higher than 70°C.^(19)^

**Figure 6:**
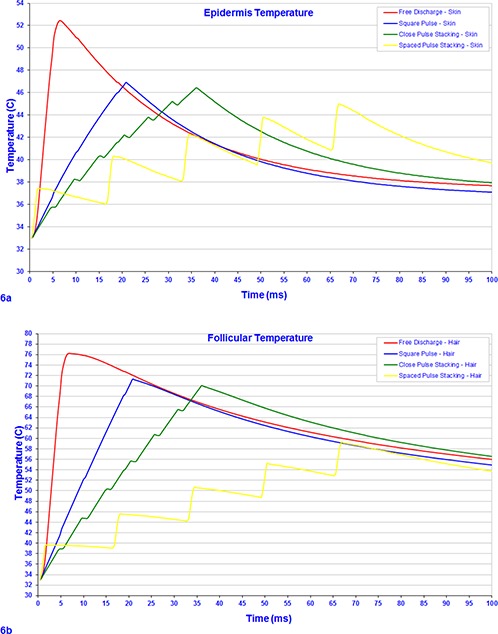
Computer modeling of the epidermis (a) using four types of commercially available temporal profiles with pulse durations of those measured in Reference (7), as depicted in The temperature profile (b) of absorbed light of the four pulse profiles using computer modeling of an *in situ* hair follicle.

The data presented in (Fig. 6a) showing skin temperatures for all four systems indicates that the peak temperature for the free discharge system produced the highest absorbed temperatures and may cause greater patient discomfort as a result (22% greater than square pulse and pulse stacking techniques).Typically, free discharge systems tend to implement active or parallel cooling of the skin to prevent adverse reactions, such as prolonged erythema or hyperpigmentation. The higher peak power dissipated into the tissue matrix of the free discharge systems may explain the greater number of adverse reactions seen with these devices in practice.^(34)^

(Figure 6b) shows free discharge, square pulse, and close pulse stacking systems to attain temperatures above 70°C within the hair follicle, which is generally considered sufficient to denature pluripotential stem cells surrounding the hair follicle and supporting regrowth. The modeling of the spaced pulse stacking system suggests that the device would need to deliver a greater radiant exposure to attain similar results than the other systems, as much energy is lost during the prolonged off time between pulses. As a result of the increased energy necessary, spaced pulse stacking systems may require more internal water cooling and active or parallel skin surface cooling to generate and deliver energy safely.

## IV. DISCUSSION

Both the square pulse and close pulse stacking temporal profiles provide sustained heating to the follicle in a controlled repeatable and consistent delivery of optical energy. This controlled delivery should also provide repeatable clinical results shot‐to‐shot. Whereas the free discharge systems, although simple in their technology, inherently vary shot‐to‐shot, and may cause disparity during treatment and during the lifetime of the device.^(35)^

The Monte Carlo model used for this study is two‐dimensional in its representation of the light‐tissue interaction. However, in reality the interaction of light with hair follicles is in three dimensions, where radial heat conduction is much faster than axial heat conduction. This may explain the retained temperatures within the temperature modeling and during exposure. The model is representative for a broad field as approximately the same amount of photons jump into as out of the azimuthal plane.

It has long been assumed that the optical properties of the various tissue layers do not change during exposure to light since the temperature increase is not sufficiently large, so constant thermal properties may be assumed. This may not be the case as during exposure, absorption of optical energy by chromophores may cause heating and mechanically modify some biological targets (for example, coagulation of blood vessels close to the surface).^36^, ^37^

The value of being able to compare the physics of light‐tissue interaction with the ability to change modeled parameters individually is of significance to clinicians and light‐based system designers. Repeatable simulations of light‐tissue interaction may be the true value of Monte Carlo modeling; however, allowance should be made for some inaccuracies compared to reality. For example, the values of absorption $\left(\mu_{a}\right)$ and scattering $\left(\mu_{s}\right)$ of tissue layers are obtained experimentally and contain an uncertainty.^(6)^ The mathematical model is a tool that can assist researchers and clinicians to identify positive theoretical models and avoid unnecessary experimental repetition. It facilitates the examination of models with hundreds of different parameters that would be practically impossible to conduct in a clinical setting. Mathematical modeling also allows investigations to be performed that cannot be conducted practically, such as viewing the deposition of photons through the skin and their final position depths in tissue.

## V. CONCLUSIONS

Monte Carlo modeling is a technique that solves the radiative transfer equation using the probabilistic nature of photon interactions and has been used to simulate many such interactions. It may be efficiently implemented for complicated tissue geometries and without restrictions in optical properties. Furthermore, this technique provides accurate results for highly absorbing and scattering media at positions close to the surface, and can effectively handle anisotropic scattering.

The computer modeling of the various pulse profiles presented here indicates that square pulse and close pulse stacking systems are efficient in delivering an optimum dose of optical radiation to induce a sufficient thermal transient in the follicular structure for effective hair reduction, in accordance with the theory of selective photothermolysis. Pulses of light that are longer than the thermal relaxation time of melanosomes, approximately 3 to 10 ms, and shorter than the thermal relaxation time for hair follicles, approximately 40 to 100 ms, will efficiently heat the hair follicle whilst the epidermis is conducting heat to surrounding tissue. Therefore, as free discharge systems have short pulse durations of circa 4–6 ms, heat has insufficient time to transfer from the epidermis during the pulse, resulting in higher peak temperature induced in the skin compared with longer pulse duration pulse types.

## References

[1] Grossman MC , Dierickx C , Farinelli W , Flotte T , Anderson RR . Damage to hair follicles by normal mode ruby laser pulses. J Am Acad Dermatology. 1996;35(6):889–94. 10.1016/s0190-9622(96)90111-5 8959946

[2] Marayiannis KB , Vlachos SP , Savva MP , Kontoes PP . Efficacy of long‐ and short pulse alexandrite lasers compared with an intense pulsed light source for epilation: a study on 532 sites in 389 patients. J Cosmet Laser Therapy. 2003;5(3–4):140–45. 10.1080/14764170310001429 14741816

[3] Chan HH , Ying SY , Ho WS , Wong DSY , Larn LK . An in‐vivo study comparing the efficacy and complications of diode laser and long pulsed Nd:YAG laser in hair removal inChinese patients. Dermatol Surg. 2001;27(11):950–54. 11737129 10.1046/j.1524-4725.2001.01048.x

[4] Roff K , Landthaler M , Hohenleutner U . Optimizing treatment parameters for hair removal using long‐pulsed Nd:YAG lasers. Lasers Med Sci. 2004;18(4):219–22. 15042427 10.1007/s10103-004-0287-9

[5] Ancona D , Stuve R , Trelles M . A multicentre trial of the epilation efficacy of a new, large spot size constant spectrum emission IPL device. J Cosmetic Laser Therapy, 2007;9(3):139–47. 10.1080/14764170701275156 17763022

[6] Ash C . Optimising output dosimetry of a broadband pulsed light source for the removal of unwanted hair [PhD Thesis]. Swansea, Wales: Swansea University; 2009.

[7] Ash C , Town G , Bjerring P . Relevance of the structure of time‐resolved spectral output to light‐tissue interaction using intense pulsed light (IPL). Lasers Surg Med. 2008;40(2):83–92. 18306158 10.1002/lsm.20596

[8] Kimel S , Svaasand LO , Hammer‐Wilson MJ , Nelson JS . Influence of wavelength on response to laser photothermolysis of blood vessels: implications for port wine stain laser therapy. Lasers Surg Med. 2003;33(5):288–95. 14677156 10.1002/lsm.10224

[9] Lucassen GW , Verkruysse W , Keijzer M , van Gemert MJ . Light distributions in a port wine stain model containing multiple cylindrical and curved blood vessels. Lasers Surg Med. 1996;18(4):345–57. 8732573 10.1002/(SICI)1096-9101(1996)18:4<345::AID-LSM3>3.0.CO;2-S

[10] Pfefer TJ , Barton JK , Smithies DJ , et al. Modelling laser treatment of port wine stains with a computer‐reconstructed biopsy. Lasers Surg Med. 1999;24(2):151–66. 10100653 10.1002/(sici)1096-9101(1999)24:2<151::aid-lsm11>3.0.co;2-0

[11] van Gemert MJ , Lucassen GW , Welch AJ . Time constants in thermal laser medicine: II. Distributions of time constants and thermal relaxation of tissue. Phys Med Biol. 1996;41(8):1381–99. 8858726 10.1088/0031-9155/41/8/009

[12] Verkruysse W , Lucassen GW , de Boer JF , Smithies DJ , Nelson JS , van Gemert MJ . Modelling light distributions of homogeneous versus discrete absorbers in light irradiated turbid media. Phys Med Biol. 1997;42(1):51–65. 9015808 10.1088/0031-9155/42/1/003

[13] Binzoni T , Leung TS , Giust R , Rufenacht D , Gandjbakhche AH . Light transport in tissue by 3D Monte Carlo: influence of boundary voxelization. Comput Methods Programs Biomed. 2008;89(1):14–23. 18045725 10.1016/j.cmpb.2007.10.008

[14] Bjerring P , Heickendorff L , Clement M , Kiernan M , Egekvist H , Patel N . The importance of temporal profile in selective non‐ablativewrinkle [oral presentation]. American Society for Lasers in Medicine and Surgery Annual Meeting. April, 2002.

[15] Steiner R , Russ D , Falkenstein W , Kienle A . Optimisation of laser epilation by simulation of the thermal laser effect. Laser Phys. 2001;2(1):146–153.

[16] Shafirstein G , Buckmiller L , Waner M , Bäumler W . Mathematical modeling of selective photothermolysis to aid the treatment of vascular malformations and hemangioma with pulsed dye laser. Lasers Med Sci. 2007;22(2):111–18. 17268765 10.1007/s10103-006-0427-5

[17] Baumler W , Vural W , Landthaler M , Muzzi F , Shafirstein G . The effects of intense pulsed light (IPL) on blood vessels investigated by mathematical modeling. Lasers Surg Med. 2006;39(2):132–39. 10.1002/lsm.20408 17066482

[18] Sun F , Chaney A , Anderson R , Aguilar G . Thermal modelling and experimental validation of human hair and skin heated by broadband light. Lasers Surg Med. 2009;41(2):161–69. 19226580 10.1002/lsm.20743

[19] Anderson RR and Parrish JA . Selective photothermolysis: precise microsurgery by selective absorption of pulsed radiation. Science. 1983;220(4596):524–27. 6836297 10.1126/science.6836297

[20] van Gemert MJ and Welch AJ . Time constants in thermal laser medicine. Lasers Surg Med. 1989;9(4):405–21. 2761336 10.1002/lsm.1900090414

[21] Raulin C , Greve B , Grema H . IPL technology: a review. Lasers Surg Med. 2003;32(2):78–87. 12561039 10.1002/lsm.10145

[22] Welch AJ and van Gemert MJ . Optical‐thermal response of laser‐irradiated tissue. New York: Plenum Press; 1995.

[23] Faber MJ , Aalders MC , Mik EG , Hooper BA , van Gemert MJ , van Leeuwen TG . Oxygen saturation‐dependent absorption and scattering of blood. Phys Rev Lett. 2004;93(2):28102. 10.1103/PhysRevLett.93.028102 15323954

[24] Manstein D , Pourshagh M , Anderson R . Effects of fluence and pulse duration for flashlamp exposure on hair follicles. Accessed on September 22, 2007 Available from: http://www.palomarmedical.com/uploaddocs/effects‐of‐fluence‐and‐pulsed‐duration‐for‐flashlamp‐exposure‐on‐hair.pdf.

[25] Saidi IS , Jacques SL , Tittel FK . Mie and Rayleigh modeling of visible‐light scattering in neonatal skin. Appl Optics. 1995;34(31):7410–18. 10.1364/AO.34.007410 21060615

[26] Wilson B and Adam G . A Monte Carlo model for the absorption and flux distributions of light in tissue. Med Phys. 1983;10(6):824–30. 6656695 10.1118/1.595361

[27] Jacques S and Wang L . Monte Carlo modeling of light transport in tissue (steady state and time of flight). In: Welch AJ and van Gemert MJC, editors. Optical‐thermal response of laser‐irradiated tissue. New York, NY: Plenum Press; 1995.

[28] Barton J , Pfefer T , Welch A , Smithies D , Nelson J , van Gemert M . Optical Monte Carlo modeling of a true portwine stain anatomy, Opt Express. 1998;2(9):391–96. 19381206 10.1364/oe.2.000391

[29] Henyey L and Greenstein J . Diffuse radiation in the galaxy. Astrophysics J. 1941;93:70–83.

[30] Crochet JJ , Gnyawali SC , Chen Y , Lemley EC , Wang LV , Chen WR . Temperature distribution in selective laser‐tissue interaction. J Biomed Opt. 2006;11:34031. 16822080 10.1117/1.2204615

[31] van Gemert M , Jacques S , Sterenborg HJ , Star WM . Skin optics. IEEETrans Biomed Eng. 1989;36(12):1146–54. 10.1109/10.42108 2606488

[32] Welch AJ and van Gemert M . Optical‐response of laser‐irradiated tissue. Holland: Kluwer Academic Publishers; 1995.

[33] Welch AJ and Gardner CM . Monte Carlo model for determination of the role of heat generation in laser‐irradiated tissue. J Biomech Eng. 1997;119(4):489–95. 9407290 10.1115/1.2798298

[34] Emerson R , Town G , Ash C , Donne K , Daniel G . Pigmentation selective photothermolysis or non specific skin necrosis using different intense pulsed light (IPL) systems [oral presentation]. American Society for Lasers in Medicine and Surgery Annual Meeting. 2008 Apr 2–6; Kissimmee, FL.

[35] Wright P , Widdowson D , Ahmed S , Shakespeare P . How well does your ruby laser work? Lasers Med Sci. 2005;20(2):104–06. 16007478 10.1007/s10103-005-0341-2

[36] Laufer J , Simpson R , Kohl M , Essenpreis M , Cope M . Effect of temperature on the optical properties of ex vivo human dermis and subdermis. Phys Med Biol. 1998;43(9):2479–89. 9755940 10.1088/0031-9155/43/9/004

[37] Jansen D , van Leeuwen T , Motamedi M , Borst C , Welch A . Temperature dependence of the absorption coefficient of water for mid‐infrared laser radiation. Lasers Surg Med. 1994;14(3):258–68. 8208052 10.1002/lsm.1900140308

